# Supplementary material for: Postsynaptic RIM1 modulates synaptic function by facilitating membrane delivery of recycling NMDARs in hippocampal neurons
Source: Nat Commun. 2018 Jun 11;9:2267. doi: 10.1038/s41467-018-04672-0 (PMC5995852; doi:10.1038/s41467-018-04672-0)
Supplement: Supplementary file 1 — Supplementary Information [file 41467_2018_4672_MOESM1_ESM.pdf]

Supplementary Information

Postsynaptic RIM1 modulates synaptic function by facilitating membrane delivery of recycling NMDARs in hippocampal neurons

Jiejie Wang[1#], Xinyou Lv[1#], Yu Wu[1], Tao Xu[1], Mingfei Jiao[1], Risheng Yang[2], Xia Li[2], Ming Chen[1], Yinggang Yan[1], Changwan Chen[1], Weifan Dong[1], Wei Yang[1], Min Zhuo[3, 4], Tao Chen[2, 3*], Jianhong Luo[1*], and Shuang Qiu[1*]

[#]These authors contributed equally to this study

*Corresponding authors. E-mail: chtkkl@fmmu.edu.cn; luojianhong@zju.edu.cn; qiushly@zju.edu.cn.

Supplementary Methods

Plasmids and antibodies

The expression vectors for GFP-tagged GluN1 (GFP-GluN1), GFP-tagged GluN2A (GFP-GluN2A), and GFP-tagged GluN2B (GFP-GluN2B) has been constructed by inserting GFP fragment in-frame with the GluN1 (NM_001270608.1), GluN2A (NM_012573.3), or GluN2B (NM_012574.1) subunit [1]. HA-tagged RIM1 (HA-RIM1) (NM_052829.1) was a gift from Ronald W. Holz (University of Michigan). GFP-tagged Rab11 (GFP-Rab11) was a gift from Dr. Weiping Zhang (Zhejiang university). GFP-RIM1 was constructed by PCR of RIM1 from HA-RIM1 and inserted into the vector pEGFP-C1. RIM1 rescue plasmid: RIM1 DNA sequence with the sequence AGAATGGACCACAAATGCTT mutated to AGGATGGATCATAAGTGTTT was inserted into the GFP-tagged RIM1 shRNA plasmid. GST-$RIM1_{1-55}$ and GST-$RIM1_{1-399}$ were constructed by PCR of amino-acids 1-55 and 1-399 of RIM1 and inserted into the vector pGEX-4T-1. The sequence 5'-AAGAATGGACCACAAATGCTT-3' was used for the suppression of endogenous rat or mouse RIM1 [2]. The nonspecific scrambled shRNA we used (5'-TTCTCCGAACGTGTCACGT-3') was from GeneChem (Shanghai, China). The sequence used for GluN2B knockdown [3]: the oligonucleotides 5'- GAT CCC CGG ATG AGT CCT CCA TGT TCT TCA AGA GAG AAC ATG GAG GAC TCA TCC TTT TTG GAA A-3' and 5'-AGC TTT TCC AAA AAG GAT GAG TCC TCC ATG TTC TCT CTT GAA GAA CAT GGA GGA CTC ATC CGG G-3' were inserted into the XhoI/BglII sites of pSUPER vector. All constructs were verified by DNA

sequencing.

Antibodies against PSD-95 (2507, 1:1000) (RRID: AB_561221) and HA (3724, 1:1000) (RRID: AB_1549585) were from Cell Signaling Technology; antibodies against synaptophysin (mAb368, 1:1000) (RRID: AB_94947), GluA1 (mAb2263, 1:1000) (RRID: AB_1977459), and GluA2 (mAb397, 1:1000) (RRID :AB_2113875) were from Millipore; antibodies against Rab3 (ab3335, 1:1000) (RRID: AB_303714), GluN2A (ab133265, 1:500) (RRID: AB_11158532), Rab5 (ab18211, 1:100) (RRID: AB_470264), PSD-95 (ab2723, 1:100) (RRID: AB_303248), Rab7 (ab5053, 1:1000) (RRID: AB_882241) and GFP (ab290, 1:1000) (RRID: AB_303395) were from Abcam; antibodies against GluN1 (sc-1467, used for IP) (RRID: AB_670215), RIM-BP2 (sc-169181, 1:500) (RRID: AB_10842119), horseradish peroxidase-linked goat anti-mouse IgG, and goat anti-rabbit IgG were from Santa Cruz Biotechnology; antibodies against RIM1 (140003, 1:1000 and 140023, 1:1000 for biochemistry) (RRID: AB_887774 and AB_2177807), RIM1 (140013, 1:500 for immunostaining) (RRID:AB_2238250), RIM2 (140303, 1:1000) (RRID: AB_2177808), Bassoon (141003, 1:1000) (RRID: AB_887697), and Munc13 (126103, 1:1000) (RRID: AB_887733) were from Synaptic Systems, and antibody against RIM1 (PAB14880, 1:1000 for biochemistry) (RRID: AB_10677359) was from Abnova; antibody against β-actin (A5316, 1:5000) (RRID: AB_476743) was from Sigma-Aldrich; antibody against mCherry (5993-100, 1:1000) (RRID: AB_1975001) was from Bio Vision; antibody against Rab11 (71-5300, 1:1000) (RRID: AB_87868) was from Invitrogen; antibody against GluN2B (AGC-003, 1:100, used for ICC) (RRID: AB_2040028) was

from Alomone; antibody against SNAP-25 (SMI 81, 1:100) (RRID: AB_510034) was from COVANCE; antibodies against GluN2B (1:500), GluN1 (1:500), and GFP (1:500) were homemade as described previously [4, 5]. The secondary antibodies for western blot were DyLight 680/800-conjugated IgG (Thermo Fisher Scientific).

Culture and transfection of hippocampal/cortical neurons and HEK293T cells

Cortical or hippocampal tissues were dissected from embryonic day 17 rats and digested in 0.25% trypsin-EDTA (Gibco) for 16 min at 37 ℃. Dissociated cells were plated onto poly-D-lysine-coated dishes and coverslips at a density of 6–10 $\times 10^4$ cells cm^{-2} in Neurobasal medium (Invitrogen) supplemented with 2% B27 (Invitrogen) and penicillin/streptomycin glutamine (Invitrogen) and kept at 37 ℃ in a 5% CO_2 humidified incubator. Half of the culture medium was replaced with fresh medium at 4 days *in vitro* (DIV4) and then at 5-day intervals. At DIV3, cytosine arabinofuranoside was added to a final concentration of 2.5 μM. Primary cultured neurons were infected with RIM1 knockdown or control lentivirus at DIV5 and used for biochemical analysis after another 7–10 days. Or, neurons were transfected with different plasmids using Ca^{2+} phosphate transfection (Clontech) at DIV6 and used for electrophysiological recording or immunostaining at DIV8-10.

Human embryonic kidney 293T (HEK293T) cells were grown in Dulbecco’s modified Eagle’s medium supplemented with 10% fetal bovine serum and antibiotics (all from Invitrogen), and transfected with specific plasmids (3-4 μg per 35-mm dish) using Lipofectamine 2000 reagent (Invitrogen) according to the manufacturer's protocol. The transfection mixture was replaced with fresh culture medium 3-5 h after

transfection. Cells were examined within 2 days after transfection. HEK293T cells were tested for mycoplasma contamination with a Bio myc Kit.

Tissue coimmunoprecipitation (co-IP)

Tissue co-IP was carried out using two different protocols. One used a protocol described previously with some modifications [4]. Cortical tissues from adult C57BL/6 mice were homogenized with 10 volumes of homogenization buffer (10 mM Tris-HCl, 320 mM sucrose, 1 mM PMSF, 1 μg mL^{-1} aprotinin, phosphatase inhibitor cocktail, pH 7.4). The homogenate was centrifuged at 700 g for 10 min at 4 ℃. A 0.10 volume of 10% sodium deoxycholate in 500 mM Tris-HCl (pH 9.0) was added to the supernatant and the preparation was incubated at 37 ℃ for 20 min. A 0.10 volume of 1% Triton X-100 in 50 mM Tris-HCl (pH 9.0) was then added, and the preparation was dialyzed against binding buffer (50 mM Tris-HCl, pH 7.4, 0.1% Triton) overnight at 4 ℃. After centrifugation (37,000 g, 4 ℃) for 15 min, IP antibody was added to the supernatant and incubated overnight at 4 ℃. Protein A-sepharose (GE Healthcare) or protein G-agarose (Thermo Fisher Scientific) was then added. After incubation for another hour at 4 ℃, the mixture was washed four times with binding buffer and eluted with 1.5 × SDS buffer by boiling at 100 ℃ for 5 min. In the other protocol, cortical tissues were lysed in lysis buffer (20 mM Tris, pH 7.5, 150 mM NaCl, 1% Triton X-100) and centrifuged (100,000 g, 4 ℃) for 1 h. IP antibodies were added to the supernatant and incubated overnight at 4 ℃.

Co-IP of transfected HEK293T cells

HEK293T cells were lysed in lysis buffer (20 mM Tris, pH 7.5, 150 mM NaCl, 1%

Triton X-100) and centrifuged (20,000 g, 4 ℃) for 15 min. IP antibodies were added to the supernatant and incubated overnight at 4 ℃. Protein A or Protein G was then added to the mixture and rotated for another 2 h under the same conditions. All subsequent procedures were similar to those for tissue co-IP.

Immunocytochemistry

All procedures were conducted at room temperature except as noted

Surface staining

Cultured neurons were rinsed 3 times with PBS, then exposed to primary antibodies for 10 min, rinsed in PBS, and incubated with Alexa 543-conjugated secondary antibodies (1:1000, Thermo Fisher Scientific) for another 10 min. After three washes with PBS, the neurons were fixed with 4% paraformaldehyde (wt/vol) in PBS for 10 min and permeabilized in PBS with 0.1% TritonX-100 and 2.5% BSA for 30 min. MAP2 antibody was applied for 1 h. After three washes with PBS, the neurons were incubated with Alexa 488–conjugated secondary antibodies (1:1000, Thermo Fisher Scientific) for another hour. After another three washes with PBS, the neurons were mounted.

Receptor internalization assay

Cultured neurons were incubated with primary antibodies (1:100, Alomone) for 10 min. After a rapid rinse with culture medium, the neurons were placed back in the incubator with original culture medium for 30 min to allow the internalization of receptors. The neurons were fixed in 4% paraformaldehyde for 10 min and incubated with blocking buffer (2.5% BSA in PBS) for 30 min. Alexa546-conjugated secondary

antibody in blocking buffer was then applied for 10 min to label the non-internalized receptors. After three washes with PBS, the neurons were permeabilized with blocking buffer containing 0.1% triton-X 100 for 30 min. The neurons were then incubated with Alexa633-conjugated secondary antibody to label the internalized population of receptors. After three washes with PBS, the neurons were mounted.

Receptor recycling assay

Cultured neurons were incubated with primary antibodies (1:100, Alomone) for 10 min. After a rapid rinse with culture medium, the neurons were returned to the incubator with original culture medium for 30 min to allow internalization of receptors. Non-internalized surface-bound antibody was then stripped by acid buffer (0.5 M NaCl and 0.2 M acetic acid) at 4 ℃ for 2 min. The neurons were returned to the incubator with original culture medium for another 30 min to allow receptor recycling back to the plasma membrane. The neurons were then fixed in 4% paraformaldehyde for 10 min and incubated with blocking buffer (2.5% BSA in PBS) for 30 min. Alexa546-conjugated secondary antibody was then applied for 10 min to label the recycled surface population of receptors. After permeabilization with blocking buffer containing 0.1% TritonX-100 for 30 min, neurons were incubated with Alexa633-conjugated secondary antibody to label the internalized population of receptors. Surface-recycled receptors and internalized receptors were labeled by Alexa546- and Alexa633-conjugated secondary antibody, respectively.

Image acquisition and analysis

Images were acquired on a confocal microscope (Fluoview FV1000; Olympus) and

analyzed with MetaMorph 7.5 software (Universal Imaging) or acquired with 3D SIM and analyzed with Imaris version 7.7.2 software (Bitplane). Gain, threshold, and black levels were not changed during individual experiments.

3D-SIM images of immunostained cells were acquired on the DeltaVision OMXV3 imaging system (Applied Precision) with a 100×1.4 oil objective (Nikon), solid-state multimode lasers (488, 593, and 633 nm) and electron-multiplying CCD (charge-coupled device) cameras (Evolve 512*512, Photometrics). Serial Z-stack sectioning was done at 125-nm intervals. The microscope was routinely calibrated with 100-nm fluorescent spheres to calculate both the lateral and axial limits of image resolution. SIM image stacks were reconstructed using softWoRx 5.0 (Applied Precision) with the following settings: pixel size 39.5 nm; channel-specific optical transfer functions; Wiener filter 0.001000; discard Negative Intensities background; drift correction with respect to first angle; and custom K0 guess angles for camera positions. Pixel registration was corrected to <1 pixel for all channels using 100-nm Tetraspeck beads. Reconstructed images were rendered in three dimensions using Imaris version 7.7.2 (Bitplane). For clarity of display, small linear changes of brightness and contrast were performed on three-dimensional reconstructions throughout the entire image.

Immunohistochemistry

Frozen sections (30 μm) were permeabilized with 0.1% Triton X-100 in PBS for 30 min at room temperature, and then blocked in 2.5% BSA for 2 h at room temperature. Sections were incubated with the antibody against RIM1 for 2 days at 4℃. After

washing with PBS, an appropriate secondary antibody and the nuclear dye DAPI were added, incubated for 4 h at room temperature, and then mounted with ProLong Gold.

GST pulldown

Bacterial lysate containing GST-RIM1$_{1\text{-}399}$, GST-RIM1$_{1\text{-}55}$, or GST protein was first incubated with GST beads (Glutathione Sepharose 4B) for 3 h at 4 ℃. After washing with PBS, 750 μl of mouse cortical lysate (containing 0.5% Triton X-100, 1 mM EDTA, 0.1 M NaCl, 0.1 mM PMSF, and 50 mM HEPES-NaOH, pH 7.4) was added to the beads and left for >12 h at 4℃ in the presence of 0.5 mM GTP-γS. The beads were then washed 5 times with PBS and resuspended in 2× loading buffer for Western blot analysis.

Preparation and infection of virus.

U6-MCS-Ubi-EGFP RIM1 shRNA lentiviral vector targeted against rat RIM1 (5'-AAGAATGGACCACAAATGCTT-3') and nonspecific control shRNA lentiviral vector were from Shanghai GeneChem, China. AAV-hSyn-Cre-EGFP and control virus (AAV-hSyn-EGFP) were from Shanghai Taitool Bioscience, China. Cultured cortical neurons were infected by lentivirus at DIV5, and the neurons were used for biotinylation detection 7 days after infection.

Surface biotinylation assay of NMDARs.

Surface biotinylation assays were conducted with cultured hippocampal neurons using an adapted protocol [6]. Primary cultured hippocampal neurons were infected with RIM1-knockdown lentivirus at DIV5. After another 7-10 days in culture, the neurons were washed twice with ice-cold PBS containing 0.5 mM $MgCl_2$ and 0.5 mM $CaCl_2$

(PBS+) and incubated with 1 mg mL^{-1} EZ-Link Sulfo-NHS-SS-biotin (Thermo Fisher Scientific) in PBS+ for 30 min at 4 ℃ with constant gentle shaking. Subsequently, the cells were washed with ice-cold quenching buffer (100 mM glycine in PBS+) and lysed in lysis buffer (1% Triton X-100 and 0.1% SDS in PBS) at 4 ℃ for 30 min. Following 15 min centrifugation at 4 ℃, the supernatant was incubated with streptavidin-Sepharose beads (Thermo Fisher Scientific) overnight at 4 ℃. The bound proteins were eluted in 1.5 × SDS buffer and assessed by Western blot analysis.

Behavioral Testing

Open field

The open field test measures hyperactivity through locomotion and anxious behavior. The apparatus consisted of a square black box (40 cm × 40 cm × 35 cm), made of Plexiglas with an outlined center area. Each animal was placed in the box and left for 15 min. Overall activity was measured by video tracking. The amount of time spent in the center area (20 cm × 20 cm) during the first 5 min as well as the total distance traveled in all areas were analyzed using Smart 3.0 software (Panlab Harvard Apparatus).

Novel Object Preference Task

Training and testing for novel object recognition was carried out as previously described [7, 8]. The box used in the open field test was also used for the novel object recognition test. Before training, mice were handled for 5 min for 5 days and then habituated to the box a day before the test, during the open field test. The activity of mice was recorded with a video camera. The task comprised an acquisition and a test

phase separated by a 4-h delay. In the acquisition phase, mice were placed in the experimental apparatus with two identical objects (referred to as A and B). At test (duration 5 min), the animal was placed in the arena again, presented with objects in the same positions as at acquisition: one object was the familiar object used at acquisition (object A) and the other was a novel object (object C). Object positions and the objects used as novel or familiar were counterbalanced. If novel object recognition memory is intact subjects spend more time investigating the novel object. The discrimination index was computed as $R(\%) = T_B*100\%/(T_A + T_B)$ or $R(\%) = T_C*100\%/(T_A + T_C)$ in the acquisition or test phase; T_A, T_B, and T_C are the times spent exploring objects A, B, and C, respectively.

Object Location Task

Training and testing for location-dependent object recognition were carried out as previously described [7, 8]. The acquisition and test phases were separated by a 4-h delay. In the acquisition phase, the mice were allowed to investigate duplicate objects (referred to as A and B) for 5 min. At test (duration 5 min), object A was placed in the same position it had occupied at acquisition while the object B was placed in the corner diagonally opposite. The position of the moved object was counterbalanced between mice. Intact object location memory is evidenced by subjects spending more time investigating the object in the new location. The discrimination index in the two phases was computed as $R(\%) = T_B*100\%/(T_A + T_B)$; TA is the time spent exploring object A, and T_B is the time spent exploring object B.

Temporal order memory task.

Training and testing for location-dependent object recognition were carried out as previously described [9]. Briefly, mice were placed in the apparatus used in the object recognition test to explore each set of three objects (referred to as A-A, B-B, and C-C) for 5 min, with a 3-min inter-session interval. After the third set of objects, the mice were given a 3-min time-out, after which a third copy of object A and a third copy of object C were placed at opposite ends of the box. The mice were then returned to the box to measure their preference for object A *versus* object C for a 5-min period. Intact temporal order memory is evidenced by subjects spending more time investigating the object that appeared early. The discrimination index was computed as R (%) = T_A * 100% / (T_A + T_C); T_A is the time spent exploring object A, and T_C is the time spent exploring object C.

Contextual Fear Conditioning

Training and testing took place in four PhenoTyper conditioning chambers as previously described [10]. Mice were placed in the conditioning chamber and received three unsignaled foot shocks (0.6 mA, 2-s duration, 30-s inter-trial interval) at 2 min after placement in the chamber. Mice were removed from the chamber after 2 min. During testing, the mice received one 5-min exposure to the conditioned context in the absence of shock 24 h after conditioning. Conditioning was assayed by measuring freezing behavior, the complete absence of movement. Freezing was scored during conditioning as well as testing.

Supplementary Figures

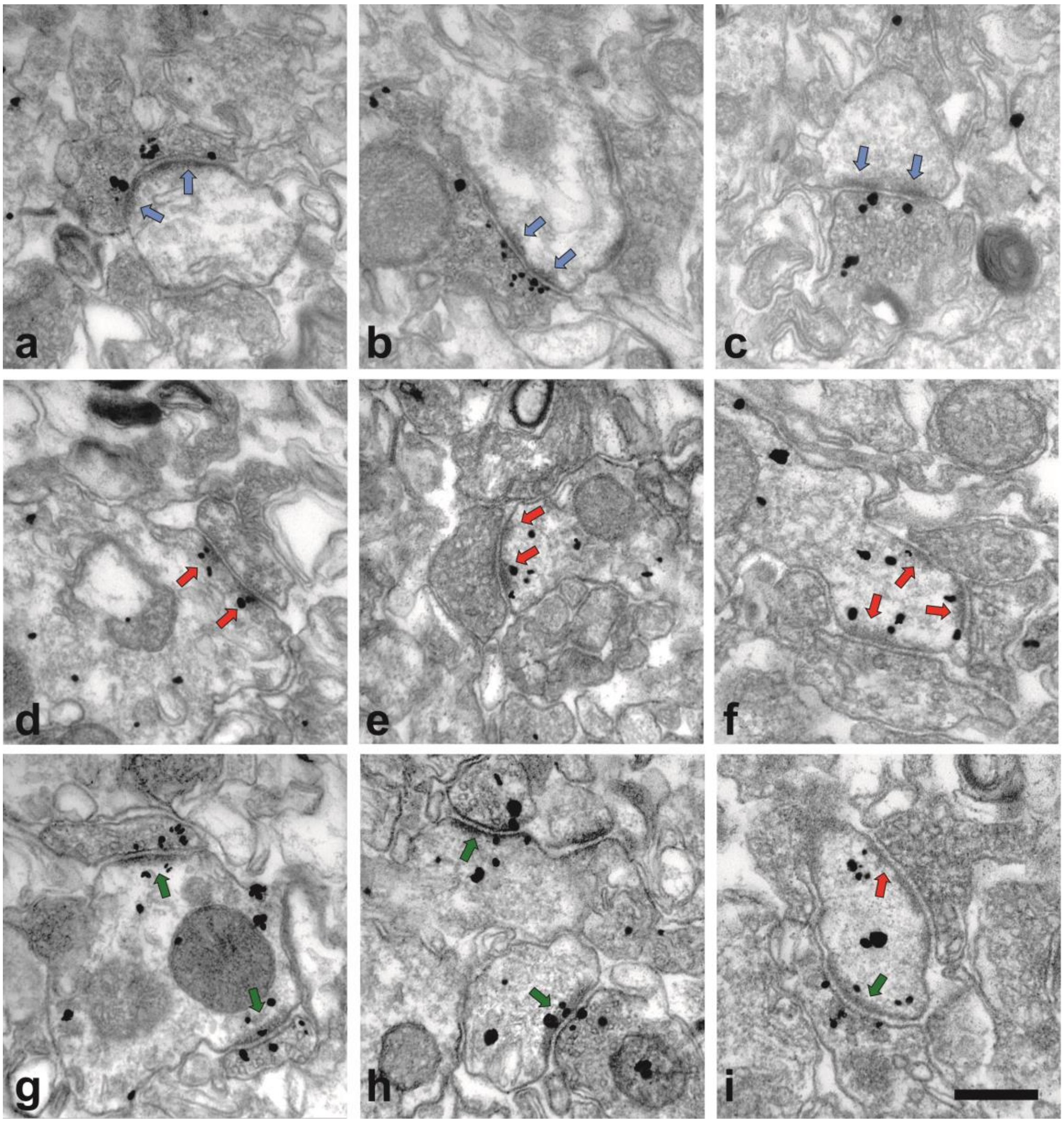

Supplementary Figure 1 Gallery of electron micrographs of RIM1 immunogold staining. (a-c) Nano-gold-labeled RIM1 distributed in presynaptic axon terminals. (d-f) Nano-gold-labeled RIM1 distributed in postsynaptic dendrites. (g-i) Nano-gold-labeled RIM1 distributed in both presynaptic and postsynaptic profiles. Blue arrows, synapses with nanogold in presynaptic profiles; red arrows, synapses with nanogold in postsynaptic profiles; green arrows, synapses with nanogold in both pre- and postsynaptic profiles. Scale bar, 500 nm in a-e and g-h; 250 nm in f and i

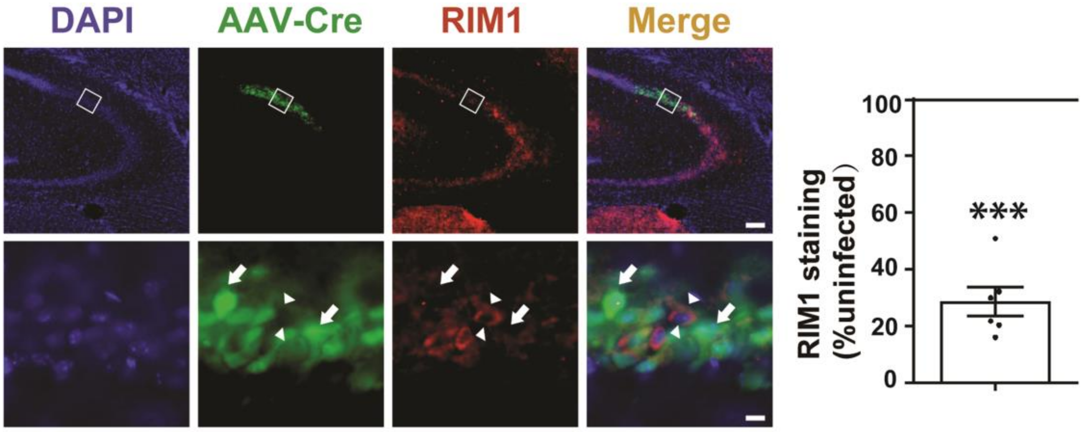

Supplementary Figure 2 RIM1 is located both presynaptically and postsynaptically. RIM1^{floxed} mice were sacrificed 3 weeks after AAV-hSyn-Cre-GFP injection. The hippocampus was sectioned and stained with antibody against RIM1 and the nuclear dye DAPI. White arrows, GFP-positive cells; arrowheads, GFP-negative cells. Scale bars, 200 μm (upper) and 20 μm (lower). t-test. ***p <0.001. Data reported in bar graphs represent mean ± s.e.m.

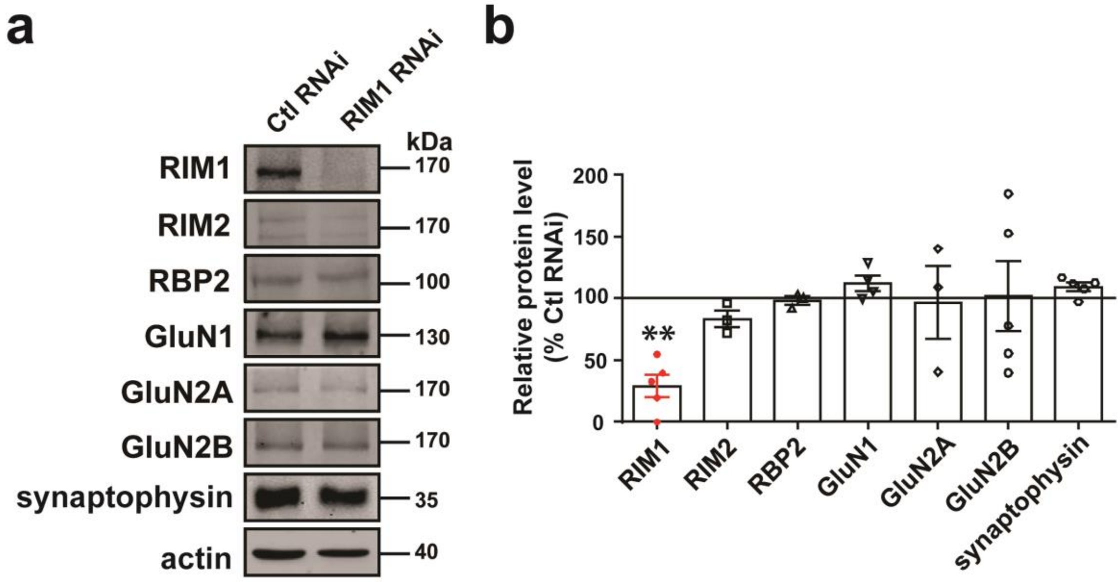

Supplementary Figure 3 RIM1 KD has no effect on the total expression of NMDARs, RIM2, or RBP2 in cultured cortical neurons. (a) Representative Western blots for the total expression levels of RIM1, NMDAR subunits, RIM2, RBP2, and synaptophysin in cultured cortical neurons infected with control RNAi or RIM1 RNAi lentivirus. (b) Statistical analysis of the expression levels of the upper proteins. n >3 independent lentivirus infection experiments from at least three different neuronal cultures. Bars represent mean ± s.e.m. t-test. ******p <0.01.

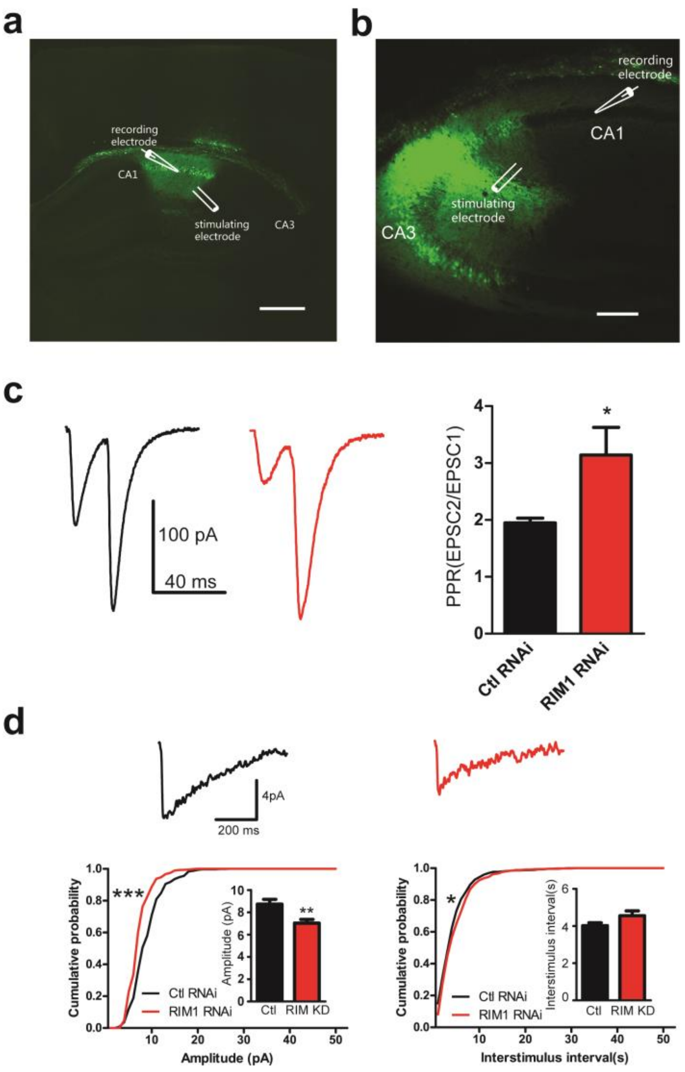

Supplementary Figure 4 Postsynaptic RIM1 KD decreases the amplitude of NMDAR-mEPSCs in the CA1 region. (a) Representative GFP fluorescence in a hippocampal slice injected with lentivirus and recording configuration in area CA1. Scale bar, 500 μm. (b) Representative GFP fluorescence in a hippocampal slice injected with lentivirus in area CA3 and recording configuration in area CA3. Scale bar, 200 μm. (c) Presynaptic RIM1 KD (RIM1 KD in area CA3) increases the paired pulse ratio of AMPAR EPSCs in the CA1 area. Left: traces of EPSCs from two representative cells. n = 9 cells from 4 mice in the control RNAi group and 10 cells

from 4 mice in the RIM1 RNAi group. Data are reported as mean ± s.e.m. t-test. *$p < 0.05$. (d) Postsynaptic RIM1 KD (RIM1 KD in the CA1 area) decreases the mean NMDAR-mEPSCs amplitude (n = 7 cells from 5 mice in the control RNAi group and 8 cells from 6 mice in the RIM1 RNAi group. Data are reported as means ± s.e.m. **$p < 0.01$, t-test) and cumulative distributions show leftward distribution shifts toward smaller mEPSC amplitudes (***$p < 0.001$, Kolmogorov-Smirnov test). Postsynaptic RIM1 KD does not affect the mean NMDAR-mEPSC inter-event intervals (data are reported as mean ± s.e.m. $p > 0.05$, t-test) while cumulative distributions show rightward distribution shifts toward larger mEPSC inter-event intervals (*$p < 0.05$, Kolmogorov-Smirnov test). Short traces of recordings from representative cells are shown above the graph.

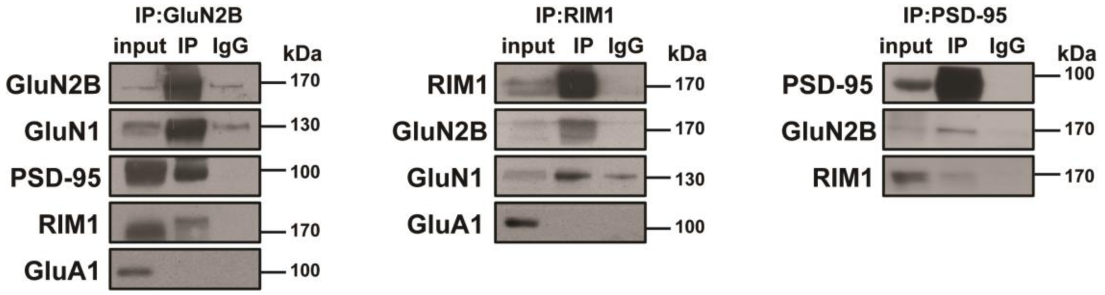

Supplementary Figure 5 RIM1, PSD-95 and NMDARs are within the same protein complexes. Extracts from adult mouse cortex were ultracentrifuged (100,000 g for 1 h) and immunoprecipitated with antibodies against GluN2B (left), RIM1 (middle), or PSD-95 (right) and blotted with corresponding antibodies. IP, immunoprecipitation.

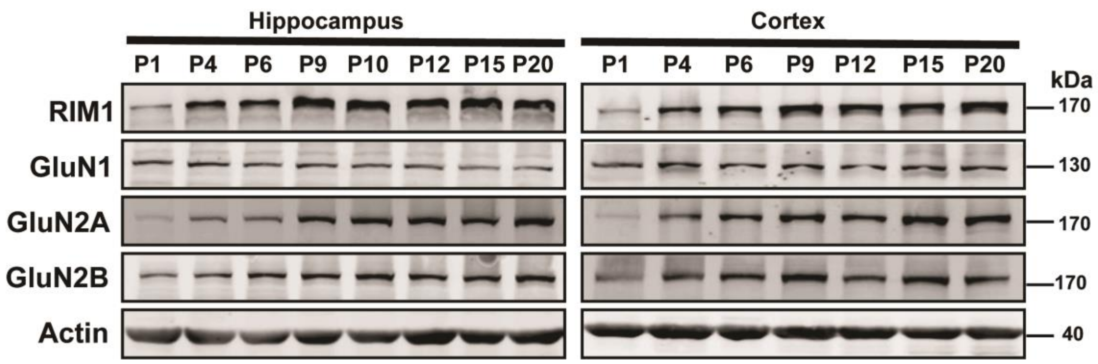

Supplementary Figure 6 Developmental map of RIM1 in rat hippocampus and cortex. Extracts of rat cortex and hippocampus from P1 to P20 were probed for RIM1, GluN1, GluN2A, and GluN2B. Expression of RIM1 gradually increases during development, as well as that of GluN2A, and GluN2B.

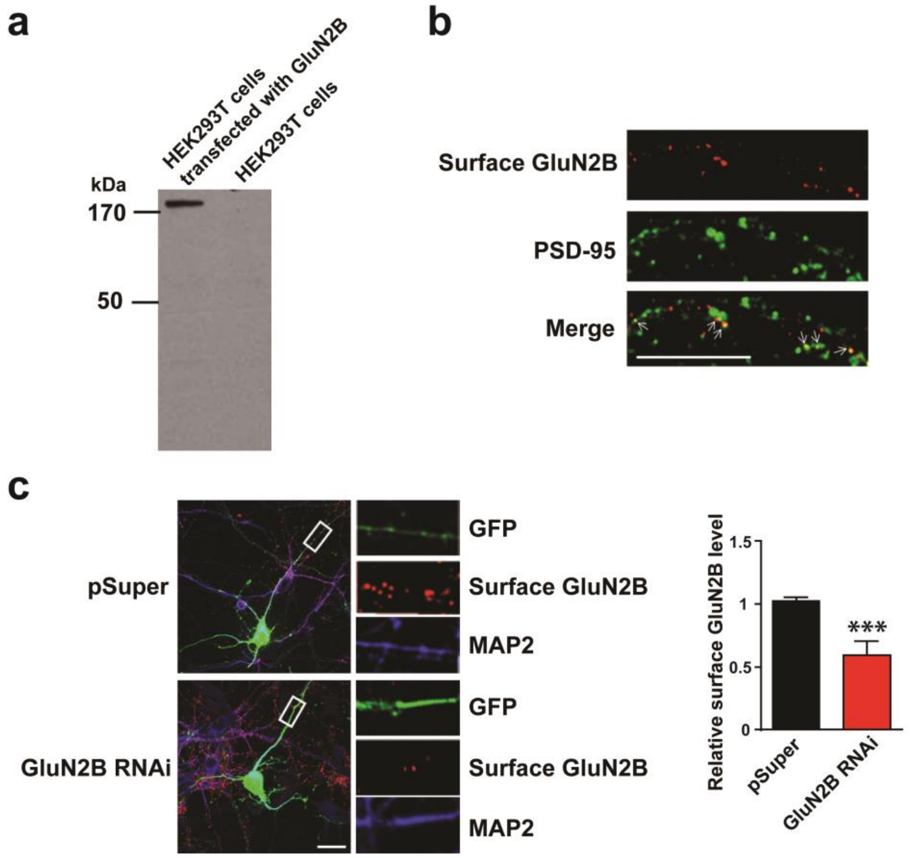

Supplementary Figure 7 Specificity of the antibody against the extracellular N-terminus of GluN2B. (a) Extracts of HEK293T cells transfected with or without GluN2B plasmids were blotted with antibody against the extracellular N-terminus of GluN2B. (b) Partial colocalization of surface-labeled GluN2B with endogenous PSD-95 in cultured cortical neurons at DIV12. Scale bar, 10 μm. (c) Surface staining of GluN2B in cultured hippocampal neurons with GluN2B knockdown. Neurons at DIV6 were co-transfected with pSuper or GluN2B RNAi plus GFP. At DIV8, neurons were surface-stained with antibody against the extracellular N terminus of GluN2B and then stained with antibody against MAP2. Scale bar, 20 μm. Bar graphs represent mean ± s.e.m. n = 16 neurons from three independent cultures. t-test. ***$p < 0.001$.

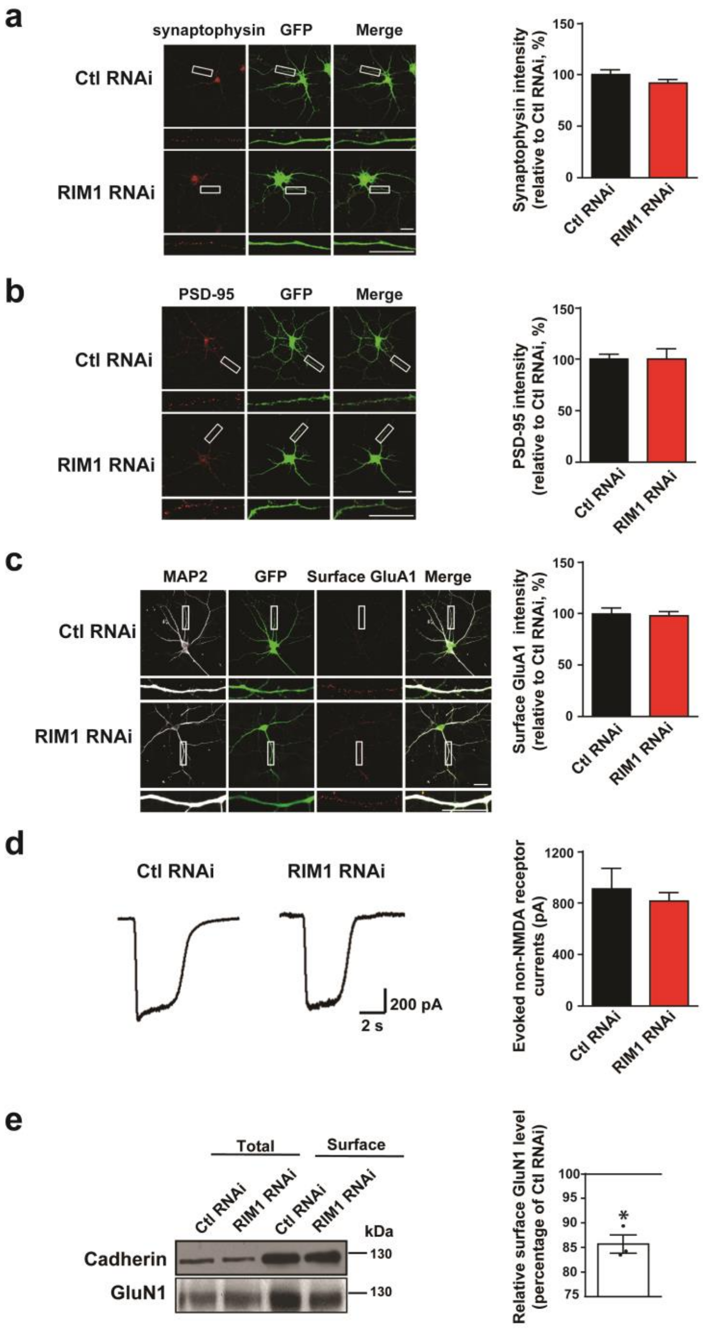

Supplementary Figure 8 RIM1 KD significantly decreases the surface expression of NMDARs analyzed by surface biotinylation. (a) Left: distribution of synaptophysin in cultured hippocampal neurons transfected with RIM1 RNAi plasmid (lower, n = 26 neurons from three independent cultures) or control RNAi plasmid (upper, n = 21 neurons from three independent cultures). Scale bar, 20 μm. Right:

statistical analysis of the cluster intensity of synaptophysin. t-test. p >0.05. (b) Left: distribution of PSD5 in cultured hippocampal neurons with RIM1 KD (lower, n = 16 neurons from three independent cultures) or without it (upper, n = 44 neurons from three independent cultures). Scale bar, 20 μm. Right: statistical analysis of the cluster intensity of PSD-95. t-test. p >0.05. (c) Left: cultured hippocampal neurons transfected with RIM1 RNAi plasmid (lower, n = 83 neurons from three independent cultures) or control RNAi plasmid (upper, n = 43 neurons from three independent cultures) were immunostained with antibodies against the N-terminus of GluA1. Scale bar, 20 μm. Right: statistical analysis of the intensity of surface GluA1 clusters. t-test. p >0.05. (d) RIM1 KD has no effect on the evoked non-NMDA currents in cultured cortical neurons. Left: representative recordings of non-NMDA currents in neurons transfected with control RNAi plasmid (n = 13 neurons from three independent cultures) and RIM1 RNAi plasmid (n = 20 neurons from three independent cultures). Right: statistical analysis of evoked non-NMDA currents. t-test. p >0.05. (e) Cultured hippocampal neurons infected with RIM1 RNAi lentivirus have lower surface expression of GluN1 than neurons infected with control RNAi lentivirus. n = 3 independent experiments from three neuronal cultures. t-test. *p <0.05.

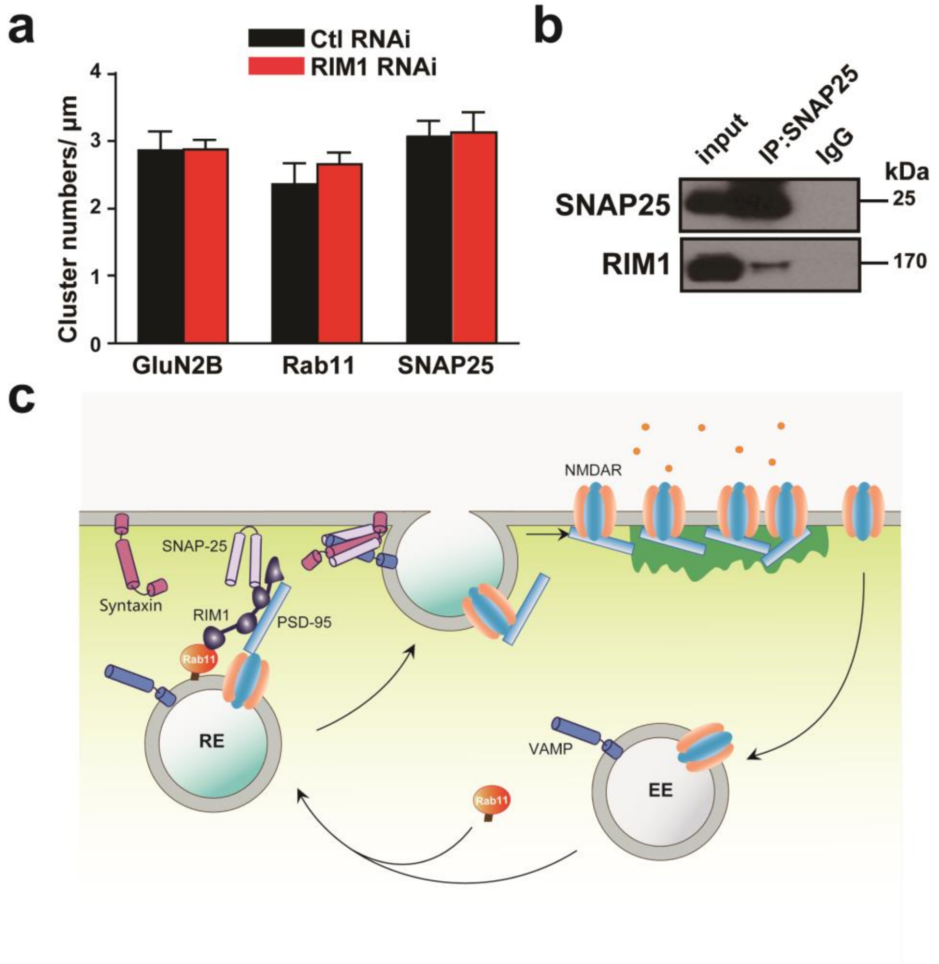

Supplementary Figure 9 RIM1 KD has no effect on the cluster density of GluN2B, Rab11, or SNAP25. (a) RIM1 KD has no effect on the cluster densities of GluN2B (control RNAi, n = 37 neurons from three cultures; RIM1 RNAi, n = 49 neurons from three independent cultures), Rab11 (control RNAi, n = 23 neurons from three cultures; RIM1 RNAi, n = 16 neurons from three independent cultures), or SNAP25 (control RNAi, n = 34 neurons from three independent cultures; RIM1 RNAi, n = 18 neurons from three independent cultures). t-test. $p > 0.05$. (b) RIM1 interacts with SNAP25 (one component of the SNARE complex) in mouse cortex. n = 3 independent experiments performed with brain tissue from three adult mice. (c)

Model of the molecular mechanisms underlying RIM1-mediated NMDAR recycling. RIM1 binds to NMDAR-containing recycling endosomes *via* interaction with Rab11. RIM1 promotes the fusion of NMDAR-containing recycling endosomes with the surface membrane by connecting directly or indirectly with the SNARE complex.

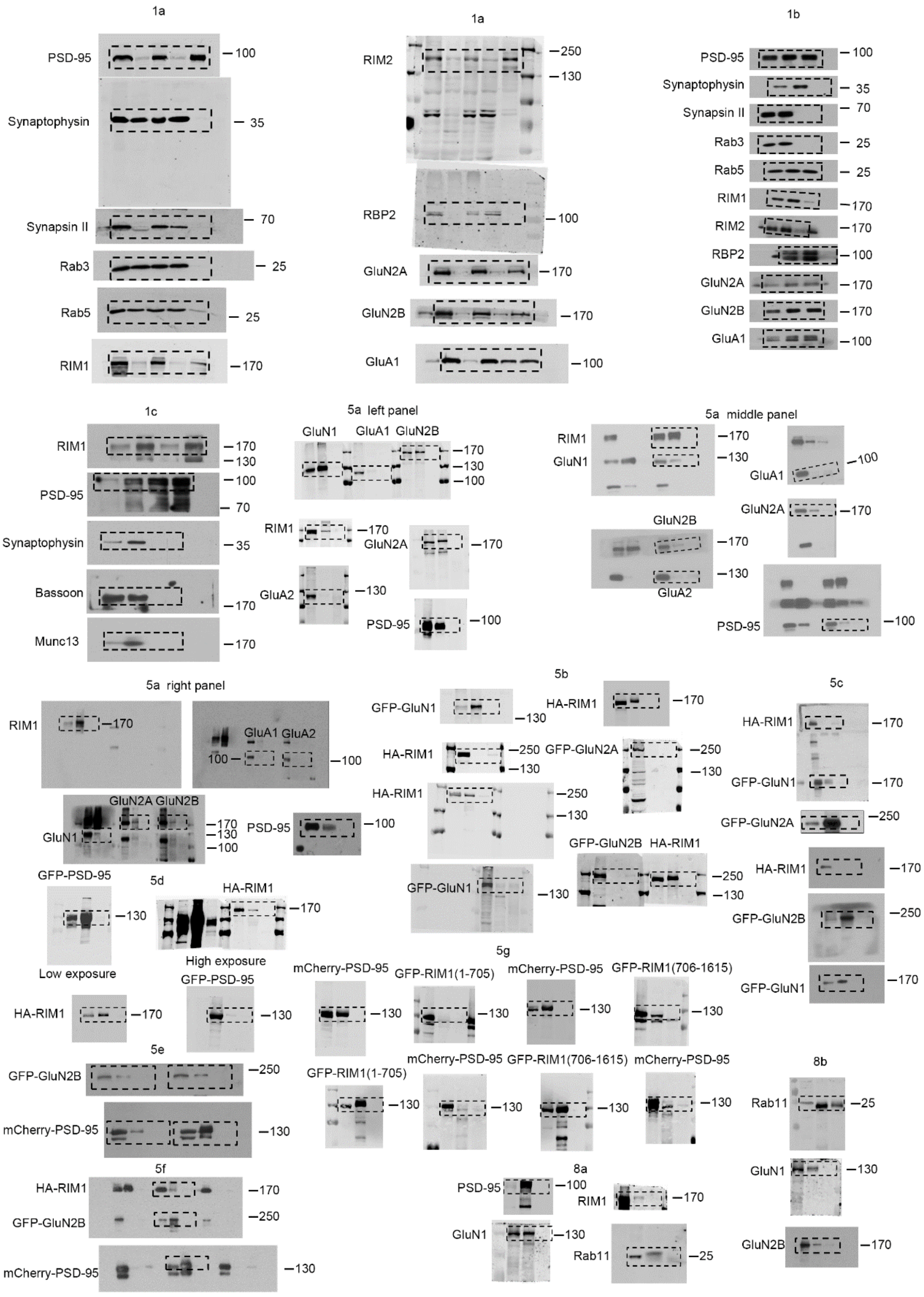

Supplementary Figure 10 Uncropped images of Western blots shown in Figures.

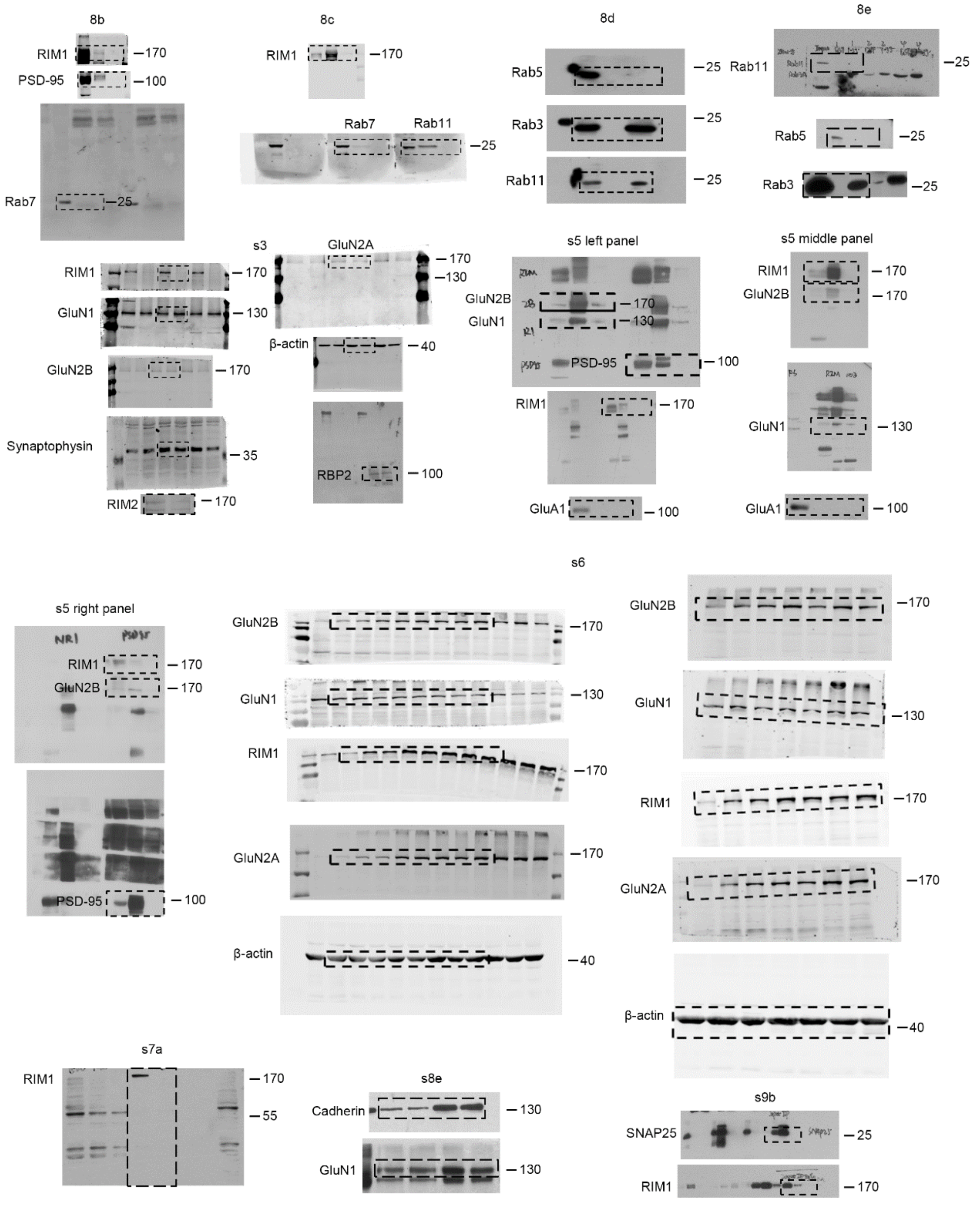

Supplementary Figure 11 Uncropped images of Western blots shown in Figures.

Supplementary References

1. Qiu S, Hua YL, Yang F, Chen YZ, Luo JH. Subunit assembly of N-methyl-d-aspartate receptors analyzed by fluorescence resonance energy transfer. *The Journal of biological chemistry* 2005, **280**(26)**:** 24923-24930.

2. Kiyonaka S, Wakamori M, Miki T, Uriu Y, Nonaka M, Bito H, *et al.* RIM1 confers sustained activity and neurotransmitter vesicle anchoring to presynaptic Ca2+ channels. *Nature neuroscience* 2007, **10**(6)**:** 691-701.

3. Kim MJ, Dunah AW, Wang YT, Sheng M. Differential roles of NR2A- and NR2B-containing NMDA receptors in Ras-ERK signaling and AMPA receptor trafficking. *Neuron* 2005, **46**(5)**:** 745-760.

4. Luo J, Wang Y, Yasuda RP, Dunah AW, Wolfe BB. The majority of N-methyl-D-aspartate receptor complexes in adult rat cerebral cortex contain at least three different subunits (NR1/NR2A/NR2B). *Molecular pharmacology* 1997, **51**(1)**:** 79-86.

5. Traynelis SF, Wollmuth LP, McBain CJ, Menniti FS, Vance KM, Ogden KK, *et al.* Glutamate receptor ion channels: structure, regulation, and function. *Pharmacological reviews* 2010, **62**(3)**:** 405-496.

6. Lu W, Fang W, Li J, Zhang B, Yang Q, Yan X, *et al.* Phosphorylation of Tyrosine 1070 at the GluN2B Subunit Is Regulated by Synaptic Activity and Critical for Surface Expression of N-Methyl-D-aspartate (NMDA) Receptors. *The Journal of biological chemistry* 2015, **290**(38)**:** 22945-22954.

7. Roozendaal B, Hernandez A, Cabrera SM, Hagewoud R, Malvaez M, Stefanko DP, *et al.* Membrane-associated glucocorticoid activity is necessary for modulation of long-term memory via chromatin modification. *The Journal of neuroscience : the official journal of the Society for Neuroscience* 2010, **30**(14)**:** 5037-5046.

8. Stefanko DP, Barrett RM, Ly AR, Reolon GK, Wood MA. Modulation of long-term memory for object recognition via HDAC inhibition. *Proceedings of the National Academy of Sciences of the United States of America* 2009, **106**(23)**:** 9447-9452.

9. van der Kooij MA, Fantin M, Rejmak E, Grosse J, Zanoletti O, Fournier C, *et al.* Role for MMP-9 in stress-induced downregulation of nectin-3 in hippocampal CA1 and associated behavioural alterations. *Nature communications* 2014, **5:** 4995.

10. Lattal KM. Effects of ethanol on encoding, consolidation, and expression of extinction following contextual fear conditioning. *Behavioral neuroscience* 2007, **121**(6)**:** 1280-1292.
